# Neuroimaging, Genetics, and Clinical Data Sharing in Python Using the CubicWeb Framework

**DOI:** 10.3389/fninf.2017.00018

**Published:** 2017-03-16

**Authors:** Antoine Grigis, David Goyard, Robin Cherbonnier, Thomas Gareau, Dimitri Papadopoulos Orfanos, Nicolas Chauvat, Adrien Di Mascio, Gunter Schumann, Will Spooren, Declan Murphy, Vincent Frouin

**Affiliations:** ^1^UNATI, Neurospin, CEA, Université Paris-Saclay, Gif-sur-Yvette, France; ^2^Logilab, Paris, France; ^3^Medical Research Council, Social, Genetic and Developmental Psychiatry Centre, Institute of Psychiatry, Psychology and Neuroscience, King’s College London, London, UK; ^4^F. Hoffmann-La Roche Pharmaceuticals, Basel, Switzerland; ^5^King’s College London, London, UK

**Keywords:** web service, data sharing, database, neuroimaging, genetics, medical informatics, Python

## Abstract

In neurosciences or psychiatry, the emergence of large multi-center population imaging studies raises numerous technological challenges. From distributed data collection, across different institutions and countries, to final data publication service, one must handle the massive, heterogeneous, and complex data from genetics, imaging, demographics, or clinical scores. These data must be both efficiently obtained and downloadable. We present a Python solution, based on the CubicWeb open-source semantic framework, aimed at building population imaging study repositories. In addition, we focus on the tools developed around this framework to overcome the challenges associated with data sharing and collaborative requirements. We describe a set of three highly adaptive web services that transform the CubicWeb framework into a (1) multi-center upload platform, (2) collaborative quality assessment platform, and (3) publication platform endowed with massive-download capabilities. Two major European projects, IMAGEN and EU-AIMS, are currently supported by the described framework. We also present a Python package that enables end users to remotely query neuroimaging, genetics, and clinical data from scripts.

## 1 Introduction

Health research strategies using neuroimaging have shifted in recent years: the focus has moved from patient care only, to a combination of patient care and prevention. In the case of neurodegenerative and psychiatric diseases, this drives the creation of increasingly numerous massive imaging studies also known as Population Imaging (PI) surveys (Hurko et al., 2012; Poldrack and Gorgolewski, 2014). It should be noticed that PI studies no longer consist of image data only. The recent wide availability of high-throughput genomics has augmented the subject data with genetics, epigenetics, and functional genomics. Likewise, the standardization of personality, demographics, and deficit tests in psychiatry facilitates the acquisition of clinical/behavioral records to enrich the subject data in large population studies. Moreover, PI studies now classically encompass more than one single imaging session per subject and cover multiple-time point heterogeneous experiments. Ultimately, these studies with complex imaging and extended data (PIx) require multi-center acquisitions to build a large target population.

A regular PIx infrastructure has to cover the following three main topics: (1) data collection, (2) quality control (QC) with data processing, and (3) data indexing and publication with controlled data sharing mechanisms. Furthermore, PIx infrastructures must evolve during the life cycle of a population imaging project, and they must also be resilient to extreme evolutions of the data content and management. In the projects we manage, we experience several extreme evolutions. The first kind of evolution may affect the published dataset such as adding a new modality for all subjects, a new time point or a new subcohort. Second, the amount of data requested evolves dramatically as the project consortium gets enlarged (Gorgolewski et al., 2015). Finally, internal ontologies have to evolve constantly in order to match the ongoing initiatives on interoperability (Scheufele et al., 2014; Gorgolewski et al., 2016).

Several existing open-source frameworks support one or several of the described topics sometimes only for one specific data type. We propose in the following a brief overview of existing systems. Some of these systems have also been reviewed by Nichols and Pohl (2015). IDA (Horn and Toga, 2009) is a neuroimaging data repository and management system that supports data collection (topic (1)) and data sharing (topic (3)). With this system, the published datasets can be searched using automatically extracted metadata. The XNAT framework (Marcus et al., 2013) is widely used for neuroimaging data and supports all the PIx infrastructure topics, focusing on tools to pipeline, and to audit the processing of image data (topic (2)). The LORIS (Das et al., 2012) and NiDB (Book et al., 2013) frameworks represent a significant effort to account for multimodal data involved in PIx studies. These frameworks, although addressing all the required topics, mainly support neuroimaging data. Openclinica (2015) and REDCap (Harris et al., 2009) facilitate the collection of electronic data such as eCRF or questionnaires and are recognized in projects of various sizes that support data collection (topic (1)). Likewise, laboratory information management systems were developed for the collection of genomic measurements such as SIMBioMS (Krestyaninova et al., 2009). Finally, the COINS framework brings essential tools for multimodal data support and, more interestingly, emphasizes the importance of providing sharing tools (topics (1) and (3)) (Scott et al., 2011).

The two European studies we manage require a tailored PIx infrastructure. Existing frameworks neither completely handle the diversity of our PIx requirements and project life cycle nor provide efficient tools to collect, check quality, and publish evolving data. Additional developments were required for building such complete infrastructure. We based these developments on a more general framework than the dedicated applications described above. In collaboration with Logilab company (Logilab SA, Paris, France), we developed three highly adaptive web services, based on the CubicWeb (CW) pure-Python framework, aimed at creating a (1) multi-center upload platform, (2) collaborative quality assessment platform, and (3) publication platform with massive-download features (Logilab, 2000). These developments were originally instituted for IMAGEN and EU-AIMS projects in order to host their data about mental health in adolescents (Schumann et al., 2010) and autism (Murphy and Spooren, 2012), respectively. The corresponding studies require key features such as upload/browse published data from the web, dynamic selection and filtering of displayed data, support for flexible download operations, high-level request language, multilevel access rights, remote data access, remote user access rights management, collaborative QC, and interoperability.

## 2 Materials and Methods

The three services described in the introduction were handled in distinct developments. Section 2.1 presents the CW framework capabilities, Sections 2.2 and 2.4 introduce the upload and publication web services through which the tailored requirements of PIx studies are satisfied. Furthermore, section 2.3 describes a collaborative rating web service that helps users to assess the data quality, and section 2.5 describes a Python API that remotely queries these web services.

### 2.1 CubicWeb Overview

**Figure 1:**
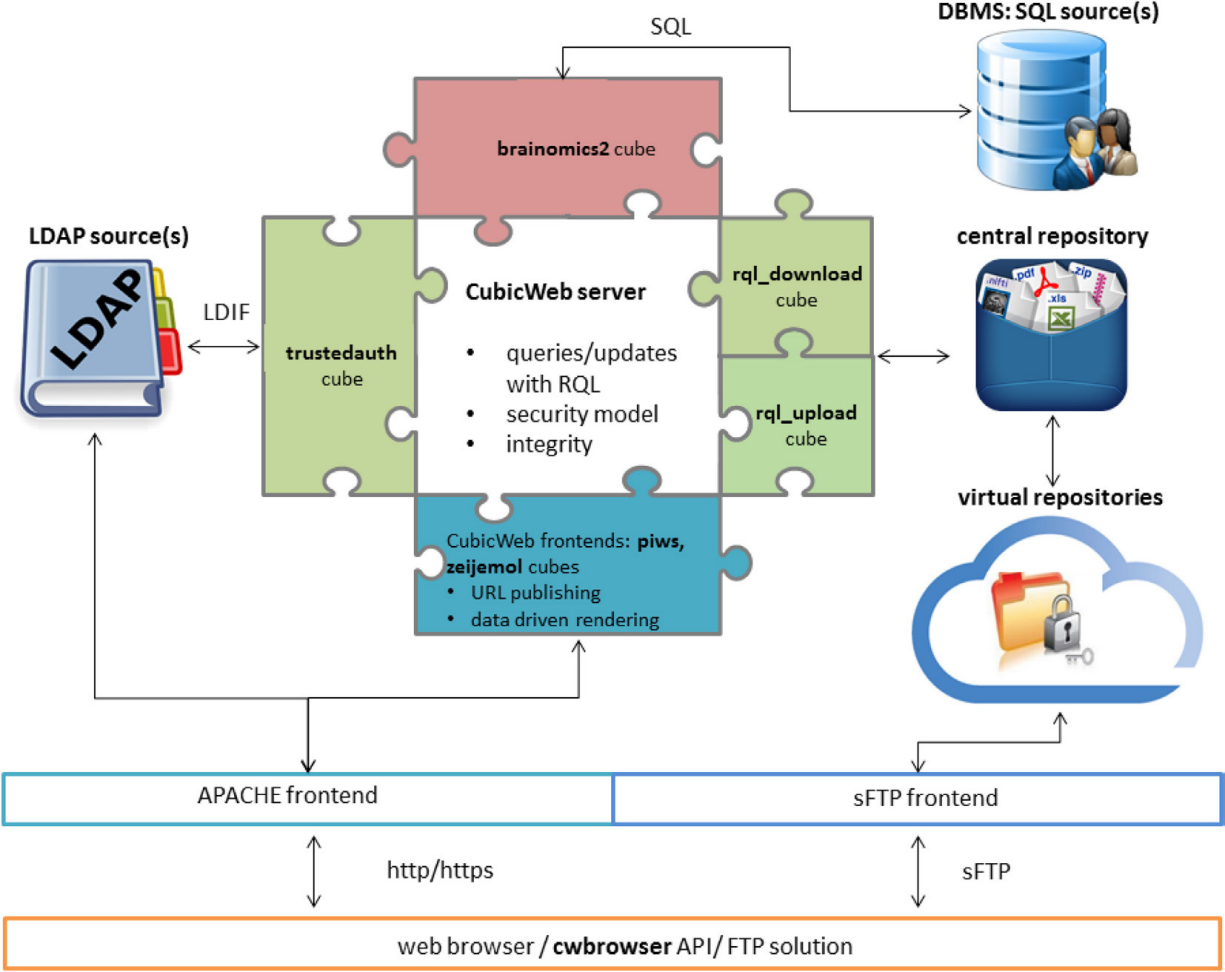
**Architecture of a CubicWeb data sharing service (DSS) integrated in an Apache platform with LDAP**. The business logic cubes provide a schema that can be instantiated in the database management system (DBMS: red puzzle piece). The system cubes ensure low-level system interactions (green puzzle piece), and the application cube proposes a web user interface (blue puzzle piece). End users access the database content through a web browser, a Python API scripting the DSS or an FTP solution, where virtual folders (acting as filters on the central repository) are proposed for download.

All the implemented services are based on the CW framework (Logilab, 2000). We choose a high level pure-Python framework that bridges web technologies and database engines. This choice was also based on the expertise and experience of people from our laboratory and a tight collaboration with Logilab (Michel et al., 2013; Papadopoulos Orfanos et al., 2015). CW distribution is organized in a core part and a set of basic Python modules, referred to as cubes, which can be used to efficiently generate web applications. The core of the CW framework, developed under the LGPL license, is constructed from well-established technologies (SQL, Python, web technologies such as HTML5 and Javascript). The main characteristics of the CW framework are given as follows:

1. CW defines its data model with Python classes and automatically generates the underlying database structure.
2. The queries are expressed with the RQL language which is similar to W3C’s SPARQL (W3C, 2013). All the persistent data are retrieved and modified using this language.
3. CW implements a mechanism that exposes information in several ways, referred to as views. This mechanism implements the classical model-view-controller software architecture pattern. Defined in Python, the views are applied to query results, and can produce HTML pages and/or trigger external processes. The separation of queries and views offers major advantages: first, the same data selection may have several web representations, and second, retrieved data can be exported in several other formats without modifying the underlying data storage.
4. All the views and triggers are recorded in a registry and are automatically selected depending on the current context, which is inferred from the type of data returned by the RQL.
5. Thanks to the semantic nature of CW, all developments inherit the possibility to follow existing or emerging ontologies, thereby facilitating sharing, access, and processing.
6. CW has a security system that grants fine-grained access to the data. This system is similar to the row-level security and policies available in the most recent versions of PostgreSQL, and links access rights to entities/relations in the schema. Each entity type has a set of attributes and relations, and permissions that define who can add, read, update, or delete such an entity and associated relations.
7. CW may run either as a standalone application or behind an Apache front server. We refer to both settings as a data sharing service (DSS) (cf. Figure 1).
8. CW can be configured to run with various database engines. For the best performance, PostgreSQL is recommended.

Starting from the basic CW distributions, our suite of services is composed of an assembly of Python modules, also referred to as cubes. The Python language is widely used in scientific communities and facilitates interfacing with major or emerging processing tools such as Nipype (Gorgolewski et al., 2011), Biopython (Chapman and Chang, 2000), Nilearn (Abraham et al., 2014), and Morphologist (Fischer et al., 2012). *Application cubes*, built over *system cubes*, and *business logic cubes* can be distinguished. The system cubes ensure interactions with the operating system and middlewares. For example, they connect to LDAP for user credentials and information or invoke FUSE (2002) as a module to construct virtual file systems in a user repository for downloading. The business logic cubes essentially provide the database schema and the application cubes define the access rights and the web interface.

Among the available Python-based frameworks, we chose CW. A major advantage of CW is the RQL language which brings end users a query interface adapted for PIx data sharing. It simplifies and improves the user experience in searching for custom datasets. RQL also avoids the use of a complicated object relational mapper (ORM), is focused on browsing relations, and allows requesting several DSS at once. The semantic nature of this request language requires the user to know only about the used data model defined as a graph (nothing about the underlying low-level relational model). This data model simplification and the expressiveness of RQL help users writing custom requests, while most of existing DSS do not expose a query language but offer a limited predefined number of operations that can be carefully designed to be efficient (e.g., RESTful APIs). Criticisms against systems exposing a query request language to the end users emphasize a risk of denial of service. To avoid this issue (i.e., overloading the server with arbitrary complex requests), CW allows limitation of usable resources (RAM per request, CPU per request, number of requests per user, CPU time per request). We believe that users should be able to select and download only what they specifically need using a query request language. This avoids filtering the data locally and saves the bandwidth.

### 2.2 Structured Data Upload Service

In PIx studies, massive and complex data are gathered from multiple data acquisition centers or devices (topic (1)). Each collected dataset must be mapped with definitions that follow consensus representation rules. Those definitions are grouped in data dictionaries that ideally follow standards (Rockhold and Bishop, 2012), but they are mainly manufacturer and/or site specific. Thus, an efficient and versatile tool is required for mapping the different data dictionaries during the collection process.

**Figure 2:**
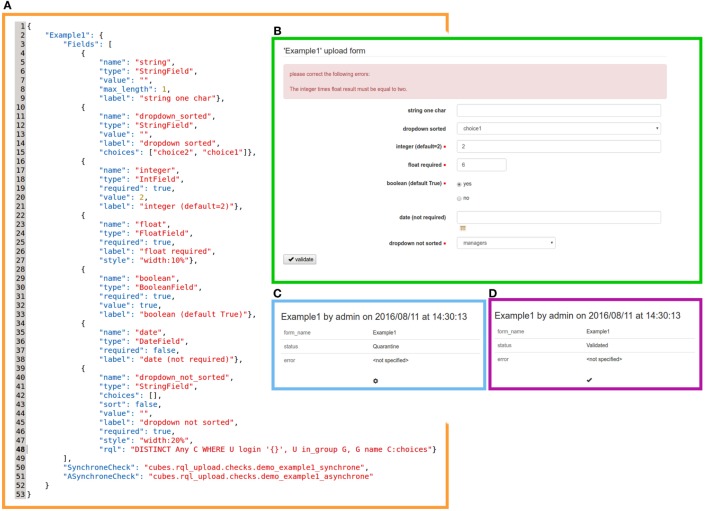
**Illustration of the upload process**. The **(A)** syntax of a form description JSON file, **(B)** corresponding web form as presented to users (here an error message returned by synchronous validation is displayed in the top red box), **(C)** “Quarantine” status, and **(D)** “Validated” status (obtained after asynchronous validation) as displayed to users: note that no feedback is shown here.

Leveraging those ideas, we implemented a flexible upload mechanism, a system cube named *rql_upload*^1^ and provided a web frontend by integrating this cube with the application cube named PIWS^2^ (Population Imaging Web Service, cf. Figure 1). Based on a CW feature that allows database completion through online HTML forms, these two cubes were developed to collect, in a DSS, both raw data and metadata. CW also enables the customization of triggers that determine the integrity of the uploaded data: synchronous and asynchronous validation filters can be specified and applied to each upload dataset. The upload proceeds as follows (cf. Figure 2):

1. Synchronous validations are applied to each form field (e.g., to check the extension of a file or the structure of an Excel table). If the validation filtering fails, then the web form is refreshed and an adapted feedback is displayed.
2. After synchronous validation, all the uploaded raw data/metadata are stored in generic entities and a “Quarantine” status is set. To avoid cluttering of the database and to ease file manipulation, files are stored in the central repository but remain accessible through the database. File hashes are automatically computed and indexed in order to assess data integrity.
3. To update the upload status from “Quarantine” to “Rejected”/“Validated,” automatic asynchronous validations can be configured in the service as looping tasks. Those validation filters are project and/or data and/or upload specific and generate adapted feedbacks for users and data managers.

Moreover, any entity or relation may be endowed with access permission rules (Logilab, 2000). Based on the CW security mechanisms, a customized security model was implemented for our upload DSS (it can be extended later). Only specific groups have the authorization to upload, and users can only access the uploads, which they are interested in. The customization of these core features allowed the creation of an upload web service that is completely described in a single JSON file. This file links the web form fields with customized or CW-internal controllers that manage the type of data to be collected.

### 2.3 Collaborative Quality Control Service

Owing to the large amount of data gathered/analyzed in PIx studies, we must consider more sophisticated operating procedures than simple quality controls (QCs), where datasets are usually only rated once by a handful of individuals. This issue can be addressed by implementing a web-based collaborative quality control process that will also remove the bias introduced by isolated raters (topic (2)). Moreover, for the studies we manage, we also added controlled vocabulary description to the ratings.

**Figure 3:**
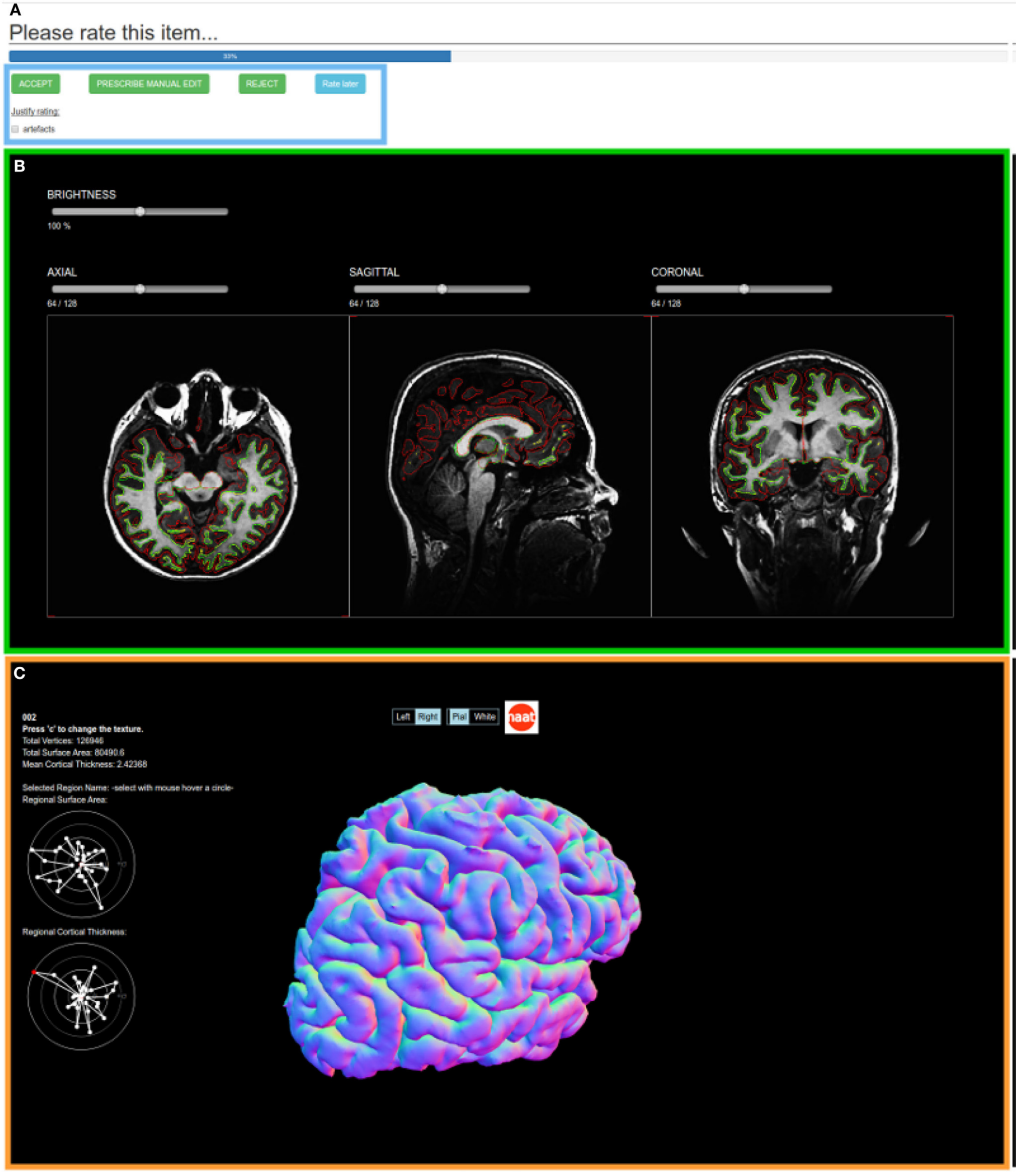
**The collaborative quality control web service of a FreeSurfer segmentation element of one subject. (A)** the quality indicators (in this case, a controlled vocabulary with an accept/prescribe manual edit/reject decision and an optional check-box justification), **(B)** a triplanar view of the white and pial surfaces overlayed on the anatomical image, and **(C)** the white and pial meshes with statistical indicators.

We achieve these goals by implementing a flexible collaborative rating mechanism, i.e., an application cube named *zeijemol*.^3^ As in section 2.2, a collaborative quality control DSS is entirely described in a single JSON file. This file consists, on the one hand, of the list of elements that will be rated (e.g., a Nifti image, a FreeSurfer segmentation, or a motion curve in a diffusion sequence of an individual) and, on the other hand, related quality indicators (e.g., binary good/bad, controlled vocabulary, scaled rating). Each element is displayed by one of the embedded viewers such as triplanar view or mesh rendering (cf. Figure 3). The QC results are stored directly in the database.

The emergence of such DSS will allow machine learning techniques to learn new classifiers to automatized the quality control task. The QC scores may also be directly used as prior knowledge during the analysis stage.

### 2.4 Publication Service

In PIx studies, data collection and QC are followed by data anonymization, ordering, and analysis. Ultimately, data are made available to the acquisition partners or the scientific community (topic (3)). While browsing the database content through the web interface, users expect to be able to download the displayed files as well as the data description and rich links between the data, also referred to as metadata. An intuitive and reliable sharing mechanism is therefore crucial as large amounts of heterogeneous evolving data must be provided. Furthermore, for the studies we manage, access rights are split along time points, scan types, questionnaires, or questions to match the consortia multilevel access permissions.

Therefore, we implemented a system cube named *rql_download*^4^ and provided a web frontend by integrating this cube with *PIWS*^5^ whenever it was used in a publication service (cf. Figure 1). The *rql_download* cube converts the result of any RQL query into files on a virtual file system that, in turn, can be accessed through a secured file transfer protocol (sFTP) (cf. Figure 1). Section 2.4.1 introduces the business logic cubes used to describe the neuroimaging genetics data and metadata and the relationship between these data. Section 2.4.2 shows how users can save the content of their current search from the DSS web interface. Section 2.4.3 describes two approaches of *rql_download*, based on two basic softwares (FUSE or Twisted), that give users access to their saved searches. This section also discusses the pros and cons of both. Section 2.4.4 presents a suitable strategy for setting user rights from the CW security system. Finally, section 2.4.5 presents a descriptive data insertion mechanism, as a set of Python scripts.

#### 2.4.1 A Dedicated Structure for Imaging Genomics Questionnaire Data

**Figure 4:**
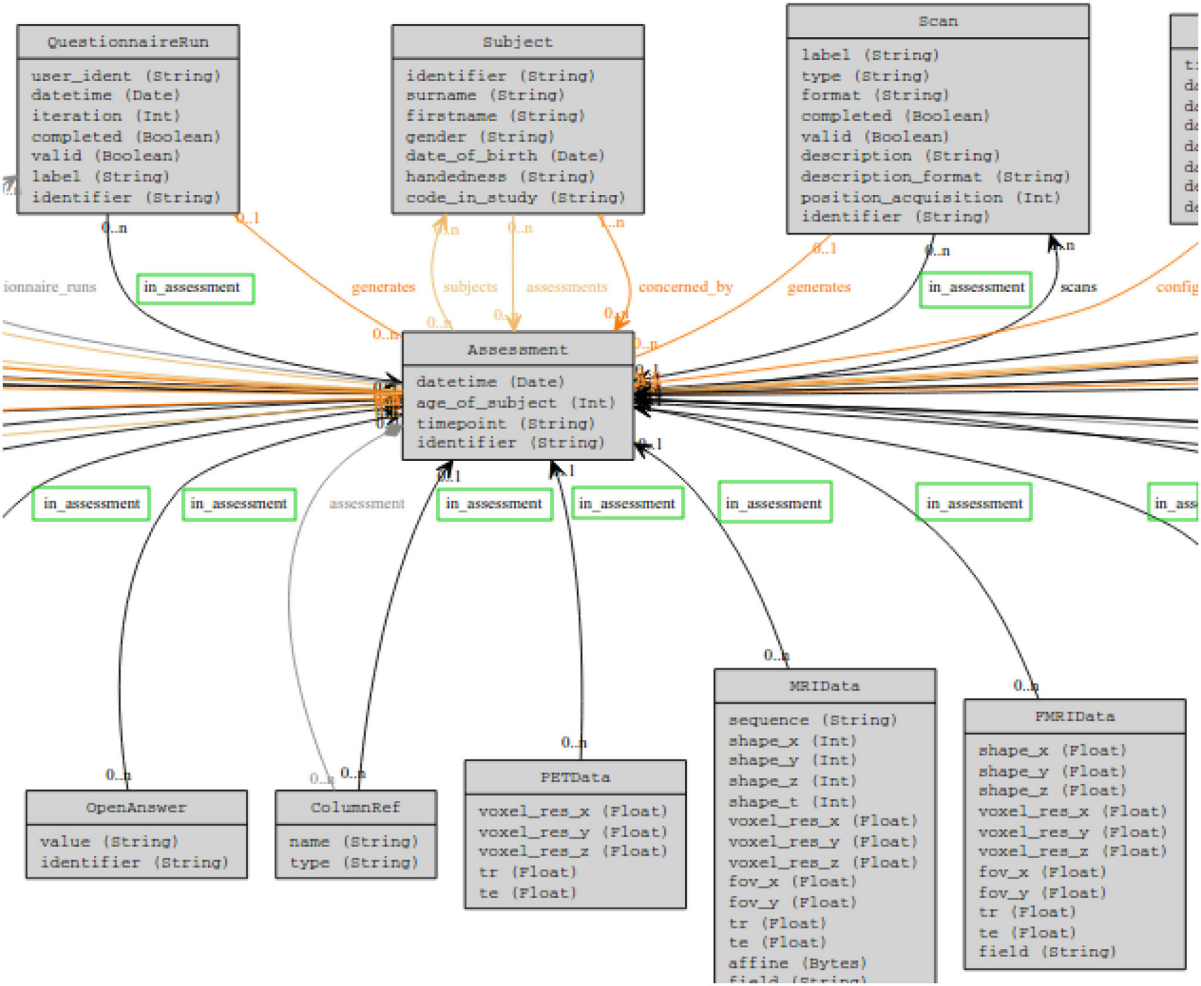
**A snippet of the schema used in a publication DSS**. We see from the green boxes that all entities are related to an “Assessment” entity through an “in_assessment” relation. This behavior is inherited from the access rights described in section 2.4.4.

The database schema was developed for handling multi-time point/multimodal datasets in the *brainomics* business logic cube.^6^ This schema supports general information such as subject data and associated metadata (age, handedness, sex, …), acquisition center definitions, multimodal imaging datasets, clinical/behavioral records, processed data, and some genomic concepts (including chromosomes, genes, SNPs, or genomic platforms). An excerpt of the produced schema is shown in Figure 4.

#### 2.4.2 Efficient Data Selection and Download Tool: The Data Shopping Cart Mechanism

**Figure 5:**
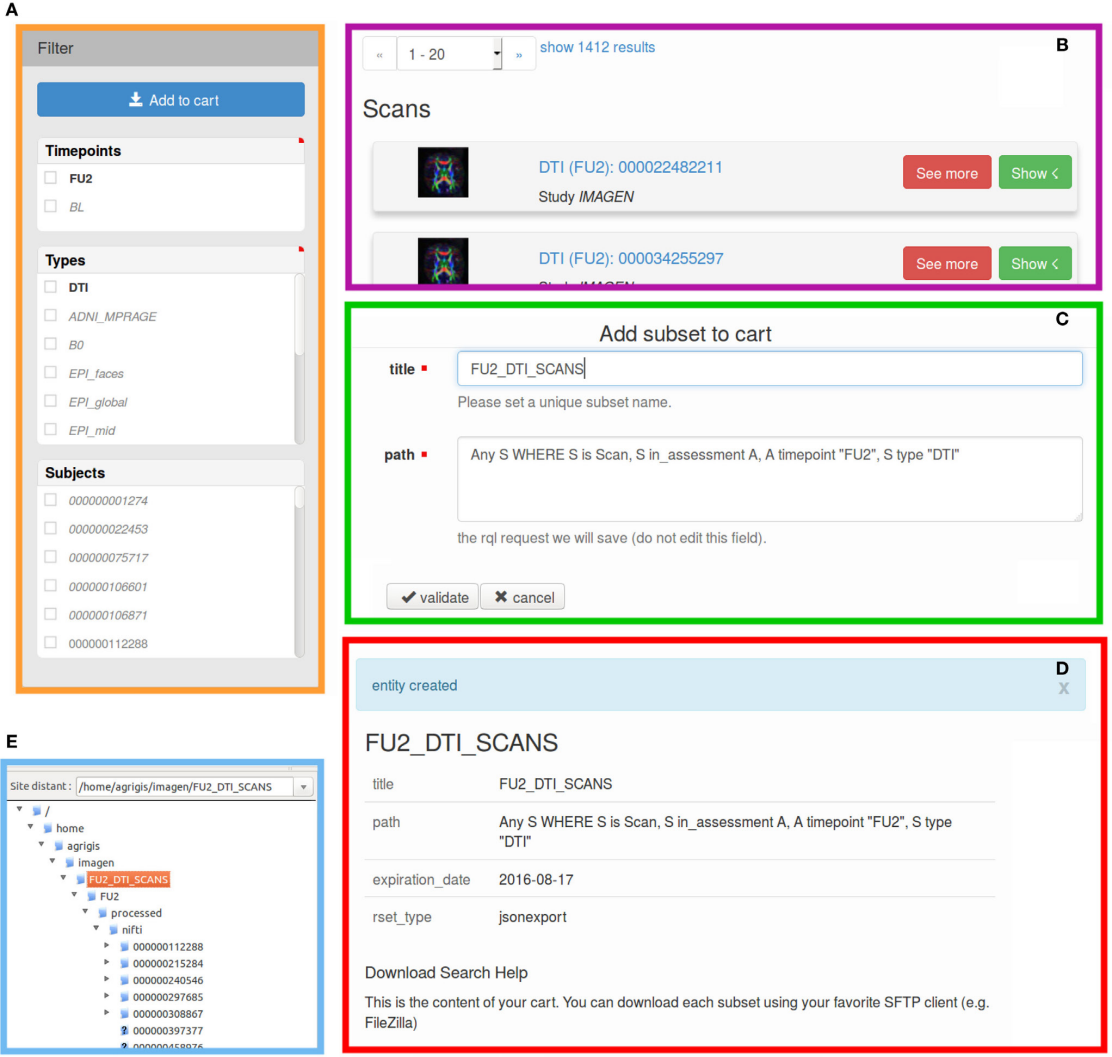
**Illustration of the download process *via* the proposed shopping cart mechanism. (A)** the facet filter bar when all the scans (“Scan” entities) are requested (as highlighted in bold, the user has selected only the “FU2” time point and the diffusion MRI “DTI” scans), **(B)** the view corresponding to the filtered dataset, **(C)** add this new search to the cart (by activating these filtering options, the save RQL path search will be automatically updated), **(D)** a new search has been created, and **(E)** the download of the search and associated files as presented in FileZilla.

When an RQL query result set is returned by the DSS, the most adapted view is automatically selected, and facets are attached to each webpage, thereby providing filtering rules. Facets allow interactive and graphical search refinements in accordance with selected attributes (e.g., sex or handedness filter for a subject result set). The developed shopping cart mechanism serves to save the user searches that consist of data, possibly large files, and metadata. This mechanism and the facet filtering are smoothly integrated: activating a filter option from the web interface automatically updates the search query result set, and thus, the list of files that will be dropped for download (cf. Figure 5). The data added to one cart has an expiration date that can be configured in the service. Convenient access rights are set: users can only access their own searches. For the sake of the EU-AIMS project hosted in our laboratory, a video explaining the data shopping cart mechanism is available.^7^

#### 2.4.3 The Transfer of the Shopping Cart Content: Data Download

When saved, the cart content is made available as virtual files and folders. A major advantage of the developed solution is that data compression or duplication is avoided, that in turn requires no extra load for the publication DSS. Data download operations are delegated to sFTP servers to ensure secure transfers. The sFTP is standard and supported by numerous client softwares on most systems.

Two approaches are implemented in the *rql_download* cube that can be selected by configuration settings:

1. *FUSE virtual folders*: For each search, the system builds a list of files to be downloaded, and subsequently creates a virtual FUSE directory acting as a filter on the central repository. The user can only see subsets of files/directories corresponding to his queries built in accordance with his access rights. Finally, the system delegates the data transfers to the sFTP server. The major advantage of this approach is the use of the standard sFTP port. However, additional system level configurations are required during the installation of the DSS in order to set the user home directories and system accounts.
2. *Twisted server*: This approach is characterized by a Python process that creates a Twisted^8^ event-driven networking server, retrieves all the searches in the database, and exposes the files *via* sFTP through the created server. Again, this process acts as a filter on the central repository where a user only sees a subset of files/directories. In this case, the authentication and file transfers are directly operated by CW. The major advantage of this strategy is that no system level configuration is required. However, listening on a non-default sFTP port, which could lead to firewall issues, is sometimes required.

#### 2.4.4 Access Rights Mechanism

In the CW security model, any entity or relation may be endowed with permission rules. To fulfill consortia’s criteria, we propose an operational setup of the CW security model for our publication DSS. We built our security model around “pivotal entities” rather than specifying rights on all entities. Pivotal entities are those on which access rights are defined, and they are related to all entities that must be covered by the security model through a specific relation (the “in_assessment” relation in Figure 4). Each time an entity covered by the security model is requested, the system automatically requests its related pivotal entity and propagates the corresponding access rights.

#### 2.4.5 The Unified Insertion Procedure

**Figure 6:**
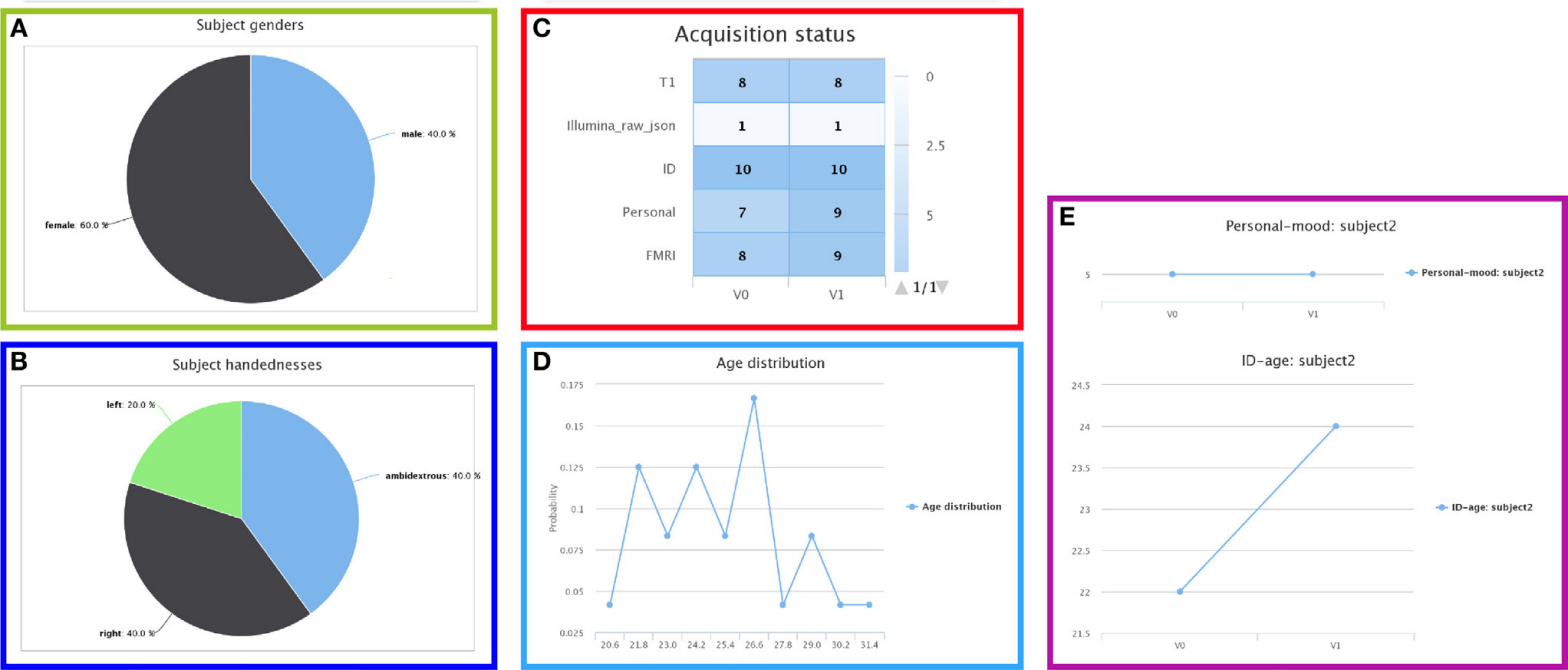
**Summary views of the database status**. Global information, for example the **(A)** gender or **(B)** handedness distributions, **(C)** acquisition status, and **(D)** age distribution, or longitudinal information, such as **(E)** the answers of subject 2 to specific questions across the study time points.

A unified insertion module is provided as a set of Python scripts to insert neuroimaging, genomic, and clinical data such as scans, genomic measures, questionnaires, and processing steps. These scripts were helpful in efficiently managing the large amount of evolving data in our projects. The indexed data are uniformly organized according to the schema structure and thus take advantage of all the aforementioned developments (e.g., shopping cart mechanism cf. section 2.4.2, security model cf. section 2.4.4, and common renderings cf. Figure 6). Generating such a DSS with these scripts can be performed without specific CW knowledge. Indeed, only a rich description of the data to be published is required as a set of Python dictionary objects.

### 2.5 A Transverse Python Module to Remotely Connect a CubicWeb DSS

With the aforementioned capabilities of the DSS, a user manually selects and downloads data through graphical interfaces in order to analyze them locally (cf. sections 2.4.2 and 2.4.3). In the case of an evolving DSS, the downloaded data must be regularly updated, and this manual process becomes time consuming and error prone when large and heterogeneous data are considered. Moreover, the metadata, such as quality scores, used to specify the dataset to download are also likely to change. Therefore, to achieve the analysis of up-to-date data stored in a DSS, direct programmatic interaction with the DSS is recommended. In the neuroimaging and neuroscience communities, data are typically analyzed by using Python scripts. Classically, the systems provide RESTful web services such as XNAT, with a Python API (Schwartz et al., 2012). Inheriting from the RQL request language, our publication DSS (cf. section 2.4) offers a rich interface to access the data.

We provide a regular Python module, named *cwbrowser*,^9^ that implements a Python API to connect and send RQL to a remote DSS based on the CW framework. This module is completely independent of CW (no CW installation required) and similar to the CW distribution *cwclientlib* cube. A publication DSS, as described in section 2.4, can be requested by the *cwbrowser* module that embeds the previously described data selection and shopping cart capabilities. It automatically fills and saves a shopping cart from a custom RQL request, downloads the associated virtual directories onto the local file system, and returns the complete requested dataset. The returned dataset contains metadata stored in the DSS such as subject sex or quality scores, and the path to the downloaded directories. These resources are organized following the DSS layout of files and folders. The users will get the same local tree which will help in writing sharable analysis scripts.

## 3 Results

Our laboratory operates several DSS for the IMAGEN project about mental health in adolescents (Schumann et al., 2010) and the EU-AIMS project about autism (Murphy and Spooren, 2012). Other DSS are currently under development to support new and ongoing initiatives. Note that the access to both IMAGEN and EU-AIMS datasets is (to date) restricted.

In the IMAGEN project, 2,000 subjects are monitored on at least two visits (the third follow-up is underway). T1, T2, FLAIR, DWI, B0, task fMRI, resting-state fMRI scans are acquired, as well as clinical/behavioral records, genotyping, gene expression, and methylation. A publication DSS at https://imagen2.cea.fr/database enables us to share more than 37,000 scans, 32,000 processing results, and 16 million distinct variables. In the near future, an upload DSS will allow us to collect a new time point.

In the EU-AIMS project, 1,500 subjects (from 6 months to 30 years old) are monitored on several visits through two distinct studies. T1, T2, FLAIR, DWI, B0, task fMRI, resting-state fMRI, and spectroscopy scans are acquired, as well as clinical/behavioral records, EEG, eye-tracking, gene expression, and methylation. An upload DSS at https://eu-aims.cea.fr/database provides the means for collecting this data from 10 centers across Europe. In addition, a collaborative quality check DSS at https://eu-aims.cea.fr/qc allows us to assess the uploaded data quality, and a publication DSS at https://eu-aims.cea.fr/data_repository enables us to share more than 13,000 scans, 12,000 processing results, and 15 million distinct variables.

## 4 Discussion and Conclusion

### 4.1 Lightweight Solution for Data Sharing

We developed a novel and lightweight PIx software infrastructure exclusively based on the CW framework. We offer a suite of CW tools that facilitates the creation of a DSS. The system delivers the data to users based on the principle of “what you see is what you get”: users define their datasets of interest by browsing the database. Thanks to the RQL and the developed Python API, remote query of a DSS is easy and intuitive. In this environment, core features such as the schema definition, the web rendering of the database content, and the semantic request language are provided by a few Python codes at the heart of the CW framework. Our DSS can use any database engine, offers an access permission mechanism, and can be smoothly integrated with the standard *Apache* environment. Moreover the CW framework relies on a large community of developers led by Logilab.

### 4.2 A PIx Swiss Knife

CW is well suited for all the scenarios one can face in a PIx project. For instance, in the projects we manage, we also provided a CW based service to allow a collaborative moderation of user access to the different DSS. This service enables the consortium review boards to assign the relevant access rights to new or existing users. It is restricted to a few members and enables the user account administration of an upload, collaborative QC, and publication DSS.

### 4.3 Future Directions

Our developments inherit the web semantic capabilities embedded in the CW framework. Thanks to this key feature, numerous problems of interoperability can be efficiently tackled using emerging ontologies and standards in neuroinformatics, neurosciences, and bioinformatics, such as the NIDM standard for data exchange (Keator et al., 2013), the Cognitive Atlas Ontology (Poldrack et al., 2011), and OntoNeuroLOG (Gibaud et al., 2011) for data annotation, or the Bio2RDF for the federation of large datasets using open-source semantic web technologies (Dumontier et al., 2014). The annotation of our datasets, with respect to these ontologies, is ongoing. Ultimately, should all DSS follow standard ontologies, RQL would provide new cross-projects querying possibilities. Although the CW framework is already used successfully in several commercial applications, it would be interesting to evaluate the CW framework performances on our DSS with Logilab dedicated tools.

## Author Contributions

AG developed the cubes, performed its deployment, and maintained the online repositories. DG, DO, TG, and RC tested the proposed application and used it in two European projects (IMAGEN, EU-AIMS). NC and AM developed the CubicWeb framework. VF, GS, WS, and DM initiated and supervized the projects. All authors contributed to the manuscript.

## Conflict of Interest Statement

The authors declare that the research was conducted in the absence of any commercial or financial relationships that could be construed as a potential conflict of interest.

## Footnotes

^1^http://neurospin.github.io/rql_upload.

^2^http://neurospin.github.io/piws.

^3^http://neurospin.github.io/zeijemol.

^4^http://neurospin.github.io/rql_download.

^5^http://neurospin.github.io/piws.

^6^https://github.com/neurospin/brainomics2.

^7^ftp://ftp.cea.fr/pub/unati/euaims/download_euaims_data.mp4.

^8^https://twistedmatrix.com/trac/.

^9^http://neurospin.github.io/rql_download/cwbrowser.

## Supplementary Material

Codes are distributed under the terms of the CeCILL-B license, as published by the CEA-CNRS-INRIA. Refer to the license file or to http://www.cecill.info/licences/Licence_CeCILL-B_V1-en.html for details. Codes are freely accessible on github https://github.com/neurospin. The DSS we are in charge of can be reached at https://imagen2.cea.fr and https://eu-aims.cea.fr.
